# Prevalence of Vitamin B12 Deficiency in Type 2 Diabetes Mellitus Patients on Metformin Therapy

**DOI:** 10.7759/cureus.37466

**Published:** 2023-04-12

**Authors:** Rathis TS, Rangabashyam Seetharaman Ranganathan, Moogaambiga Solai Raja, Pillutla Sai Sareen Srivastav

**Affiliations:** 1 General Medicine, Vinayaka Mission Kirupananda Variyar Medical College and Hospital, Salem, IND; 2 Internal Medicine, Vinayaka Mission Kirupananda Variyar Medical College and Hospital, Salem, IND

**Keywords:** “ metformin” “antidiabetic drugs”, from india, causes of vitamin b12 deficiency, type-2 diabetes mellitus, metformin therapy

## Abstract

Background

Long-term metformin treatment in individuals with type 2 diabetes mellitus causes vitamin B12 insufficiency, which is typically neglected, undetected, and under-treated. A severe deficit may cause life-threatening neurological problems. This study assessed the prevalence of vitamin B12 deficiencies among T2DM patients and its factors at a tertiary hospital in the Tamil Nadu district of Salem.

Materials and Methods

This is an analytical cross-sectional study conducted in a tertiary care hospital in the Salem district, Tamil Nadu, India. Patients with type 2 diabetes mellitus who were prescribed metformin at the outpatient department of general medicine took part in the trial. Our research instrument was a structured questionnaire. We used a questionnaire containing information on sociodemographic characteristics, metformin use among diabetic mellitus patients, diabetes mellitus history, lifestyle behaviors, anthropometric measurement, examination findings, and biochemical markers. Prior to administering the interview schedule, each participant's parents provided written informed consent. A thorough medical history, physical exam, and anthropometric examination were performed. Data were entered in Microsoft Excel (Microsoft Corporation, Redmond, WA) and analyzed using SPSS version 23 (IBM Corp., Armonk, NY).

Results

Among the study participants, we diagnosed nearly 43% of diabetes cases in participants between the ages of 40-50 years, while we diagnosed 39% aged under 40 years. Nearly 51% had diabetes for 5-10 years, while only 14% had diabetes for over 10 years. In addition, 25% of the study sample had a positive family history of type 2 diabetes. Nearly 48% and 13% of the study group had been on metformin for 5-10 years and >10 years, respectively. The majority, 45%, were found to take 1000 mg of metformin per day, whereas just 15% take 2 g per day. In our study, the prevalence of vitamin B12 insufficiency was 27%, and nearly 18% had borderline levels. The duration of diabetes mellitus, the duration of metformin intake, and the dose of metformin were statistically significant (p-value = 0.05) among the variables associated with diabetes mellitus and vitamin B12 deficiency.

Conclusion

The results of the study show that a deficiency in vitamin B12 increases the likelihood that diabetic neuropathy would worsen. Therefore, individuals with diabetes who take larger dosages of metformin (more than 1000mg) for an extended period must have their vitamin B12 levels monitored often. Preventative or therapeutic vitamin B12 supplementation can mitigate this issue.

## Introduction

Hyperglycemia is a symptom of the metabolic disorder diabetes mellitus, which is characterized by difficulties in the breakdown of carbohydrates, lipids, and proteins. Patients with diabetes are deficient in absolute or relative insulin. Diabetes mellitus is a significant noncommunicable disease whose prevalence has exploded during the past two decades [1]. In the technologically advanced world of the twenty-first century, diabetes mellitus has arisen as a significant threat to public health, and with time it has become an epidemic that cannot be ignored [2,3].

According to a recent report by the World Health Organization (WHO), India has the highest number of diabetics in the world, surpassing China [4]. Diabetes mellitus affects around 88 million Southeast Asians, a number that is projected to rise to 153 million by 2045. Diabetes mellitus affects around 77 million people in India between the ages of 20 and 79, accounting for 8.9% of the population [5].

 In the "biguanide" class, metformin is an anti-hyperglycemic medication. Metformin decreases blood glucose levels and is the cornerstone of treatment for type 2 diabetes [6]. Metformin decreases the insulin resistance characteristic of T2DM by enhancing insulin sensitivity. If glycemic control is insufficient, we may use metformin alone or in combination with other oral hypoglycemic medications [7].

Cobalamin is an important B vitamin that is water soluble. Vitamin B12 plays a significant role in normal brain function (both central nervous system and peripheral nervous system), DNA synthesis, and red blood cell (RBC) synthesis [8]. Vitamin B12 is naturally abundant in non-vegetarian diets, and the recommended daily requirement (RDA) is 2.4 micrograms per day. A normal vitamin B12 concentration exceeds 221 pmol/L in the human body [9]. Nearly 50% of subclinical vitamin B12-deficient patients have normal vitamin B12 levels [10].

Metformin inhibits calcium-dependent channels in the ileum, hence preventing the absorption of vitamin B12 in the ileum. Several studies have shown that extended use of metformin causes vitamin B12 insufficiency through this mechanism [11-16]. A severe deficit may cause significant neurological problems [17]. According to the literature, 30% of T2DM patients on long-term metformin exhibited vitamin B12 malabsorption, and multiple studies conducted in various regions of the world suggest that metformin medication reduces serum vitamin B12 levels by 14% to 30% [18-21].

The literature on vitamin B12 deficiency is also devoid of findings, and they have undertaken few investigations on the frequency of vitamin B12 deficiency in Tamil Nadu. This study assessed the frequency of vitamin B12 deficiencies among T2DM patients and its causes at a tertiary hospital in the Tamil Nadu district of Salem.

## Materials and methods

Study setting and study period

We conducted this study as an analytical cross-sectional study in a hospital of tertiary care in the Tamil Nadu district of Salem. Participants in the study were type 2 diabetics using metformin who visited the General Medicine outpatient department. We conducted the study from November 2020 to April 2021, six months.

Sample size estimation

We estimated the sample size based on a 2012 study by Reinstatler et al. in the United States, which found a prevalence of vitamin B12 deficiency of 5.8% [22]. We calculated the sample size using the formula, N = 3.84*p*q/ d2. The sample size was 83, allowing for a non-response rate of 15%. We computed the final sample size to be 96, rounded up to 100.

Selection criteria

The inclusion criteria were patients having type 2 diabetes for at least two years, aged between 30 and 75 years, free of urinary tract infection complaints, taking metformin up to the maximum daily dose of 2,000 mg, and willing to participate in the study. Patients with type 1 diabetes, type 2 diabetes mellitus patients on insulin therapy or mixed therapy (insulin with metformin or other hypoglycemic drugs), pregnant women, patients with pernicious anemia or a history of malabsorption syndrome after gastrointestinal surgery, patients with chronic hepatitis, and those with hypothyroid disorders were excluded from the study.

Ethics clearance

Before conducting the research, we got approval from the Institutional Ethics Committee of Medical College and Hospital (No: VMKVMC&H/ IEC/20/08). The informed consent was drafted in the local language and was based on ICMR criteria. Before beginning the study, we received approval from our institution's Ethics Committee.

Data collection procedure

We adopted a semi-structured questionnaire to get data from study participants through interviews. We used a questionnaire containing information on sociodemographic characteristics, metformin use among diabetes mellitus patients, diabetes mellitus history, lifestyle behaviors, anthropometric measurement, examination findings, and biochemical markers. After receiving consent, a single investigator performed the interview, and their responses were documented on the questionnaire. Each participant was interviewed for 10 to 15 minutes. A thorough medical history was taken and after collecting blood samples, biochemical analyses were conducted.

Data analysis

Data were entered in Microsoft Excel (Microsoft Corporation, Redmond, WA) and analyzed using SPSS v. 23 (IBM Corp., Armonk, NY). Descriptive and analytic statistics were used for our data analysis. We carried out a descriptive analysis and presented qualitative data as frequencies and percentages as well as means and standard deviations for qualitative data. The odds ratio was determined, and the chi-square test was employed for statistical analysis (a p-value of less than 0.05 is considered as significant).

## Results

About 47% of samples were of patients aged 40-50 years, while only 12% were over the age of 60. The respondents' average age was 49.93 ± 9.58 years. In our survey, there was a preponderance of men (55%), and only 45% were females. Around 16% of the population was illiterate, while 31% had completed middle school and 22% had completed high school. Regarding occupation, 25% were unemployed, while 40% and 24% were engaged in unskilled and semi-skilled occupations, respectively (Table 1).

**Table 1:** Sociodemographic characteristics of the study respondents (N=100)

Variables		Frequency (%)
Age	< 40 Years	14(14%)
	40-50 Years	47(47%)
	51-60 Years	27(27%)
	> 60 Years	12(12%)
Gender	Male	55(55%)
	Female	45(45%)
Education	Illiterate	16(16%)
	Primary school	24(24%)
	Middle school	31(31%)
	High school	22(22%)
	UG/PG	7(7%)
Occupation	Unemployed	25(25%)
	Unskilled	40(40%)
	Semi-skilled	24(24%)
	Skilled	6(6%)
	Farmers/shop owners	5(5%)

About 22% of the individuals in the study were smokers, while 37% of the participants consumed alcohol regularly. Only 12% were hypertensive, 6% had chronic obstructive pulmonary disease (COPD), and 4% had coronary artery disease.

Around 43% of diabetic patients were diagnosed between the ages of 40 and 50, whereas 39% of diabetic patients were diagnosed before the age of 40. Nearly half had diabetes mellitus for 5-10 years, while only 14% had diabetes for beyond 10 years. In addition, 25% of the study sample had a positive family history of type 2 diabetes. Estimated 48% and 13% of the study group had been on metformin for 5-10 years and > 10 years, respectively. Around 45% of patients were taking 1000 mg of metformin per day, whereas just 15% were taking 2 g per day. About 45% of responders on metformin report taking 500 mg twice daily, while 39% were taking 500 mg three times daily.

In our study, 27% of participants had vitamin B12 deficiency, approximately 18% had borderline levels, and the mean vitamin B12 level was 308.6 ± 6115.58 pmol/L. Around 27% of respondents were deficient in vitamin B12 (148 pmol/Litre), whereas 55% were normal (> 221 pmol/litre) (Table 2). 

**Table 2:** Variables pertaining to lifestyle, diabetes mellitus and vitamin B12 (N=100)

Variables		Frequency (%)
Smoking history	Yes	22(22%)
	No	78(78%)
Alcohol consumption	Yes	37(37%)
	No	63(63%)
Comorbidities	Hypertension	35(12%)
	COPD	17(6%)
	Anaemia	11(4%)
	CAD	11(4%)
	Others	23(8%)
Age of onset of diabetes mellitus	< 40 Years	39(39%)
	40-50 Years	43(43%)
	51-60 Years	12(12%)
	> 60 Years	6(6%)
Duration of diabetes mellitus	< 5 Years	35(35%)
	5-10 Years	51(51%)
	> 10 Years	14(14%)
Family history of diabetes mellitus	Yes	25(25%)
	No	75(75%)
Metformin duration	< 5 Years	39(39%)
	5-10 Years	48(48%)
	> 10 Years	13(13%)
Metformin dose (mg/day)	500	1(1%)
	1000	45(45%)
	1500	39(39%)
	2000	15(15%)
HbA1c	<7	59(59%)
	>7	41(41%)
Vit B 12 deficiency	Normal	55(55%)
	Borderline	18(18%)
	Deficiency	27(27%)
Vit B 12 values	<148pmol/ liter	27(27%)
	148-221 pmol/ liter	18(18%)
	>221 pmol/ liter	55(55%)
Anaemia	Yes	57(57%)
	No	43(43%)

In our study, the duration of diabetes mellitus, duration of metformin intake, and dose of metformin were statistically significant (p-value <0.05) among the variables related to diabetes mellitus and vitamin B12 deficiency. We described the association between variables related to diabetes mellitus and vitamin B12 deficiency among the study respondents in Table 3.

**Table 3:** Association between variables related to diabetes mellitus and vitamin B12 deficiency among the study respondents (N=100) *Statistically significant association since the p-value is less than 0.05

Variables related to diabetes mellitus		Vitamin B12 deficiency			Chi-square value	p-value	Odds ratio (95% CI)
		Yes (27)	No (73)				
Age of onset of DM	≥ 45 Years	10		27	0.000	0.966	0.98 (0.39-2.45)
	< 45 Years	17		46			
Duration of DM	≥ 7 Years	25		20	33.849	< 0.0001*	33.12 (7.17-152.88)
	< 7 Years	2		53			
Presence of comorbidity	Yes	8		26	0.314	0.575	0.76 (0.29-1.97)
	No	19		47			
Duration of metformin use	≥ 7 Years	24		16	36.834	< 0.0001*	28.50 (7.59-106.91)
	< 7 Years	3		57			
Dose of metformin (mg/day)	> 1000	22		32	11.245	0.001*	5.63 (1.92-16.52)
	≤ 1000	5		41			
Glycemic control	Yes	15		44	0.181	0.670	0.82 (0.33-2.20)
	No	12		29			

In our study, variables significantly correlated with serum vitamin B12 values were duration of diabetes mellitus (p-value = < 0.0001, r=-0.501), duration of metformin use (p-value = < 0.0001, r = -0.507), daily dose of metformin (p-value = 0.0004, r = -0.342), and hemoglobin (p-value = < 0.0001, r = 0.931). We found no significant correlation for other variables (p-value = > 0.05). We showed the correlation between serum vitamin B12 value and study variables in Table 4.

**Table 4:** Correlation between serum Vitamin B12 value and study variables * Pearson’s correlation test was used at 0.01 level of significance (two-tailed)

Variables	Correlation coefficient (r)	p-value
Age	- 0.096	0.342
Age of onset of diabetes mellitus	0.133	0.188
Duration of diabetes mellitus	- 0.501	< 0.0001*
Duration of metformin use	- 0.507	< 0.0001*
Daily dose of metformin	- 0.342	0.0004*
BMI	0.137	0.173
Hemoglobin	0.931	< 0.0001*
HbA1c	0.133	0.187
Serum creatinine	- 0.127	0.209

## Discussion

In our study, the mean age of the respondents was 49.93 ± 9.58 years. The mean age was comparable to our study in studies by Raizada et al., Agarwal et al., Baidya et al., Miyan et al., and Aroda et al., in which the mean age was 50.1 ± 11.5 years, 51.98 ± 5.17 years, 51.37 ± 4.49 years, 51.16 ± 14.64 years, and 51.2 ± 10.1 years, respectively [12,23-26].

We have observed male preponderance (55%) in our study, and females made up only 45%. Similarly, we noted male preponderance in studies by Reinstatler et al., Baidya et al., Chattopadhyay et al., and Singh et al., in which males made up 50.3%, 52%, 57%, and 57.1%, respectively [22,24,27,28].

In our study, the mean duration of metformin medication was 6.42 ± 3.37 years, whereas, in studies by Ahmed et al., Nervo et al., Sato et al., Baidya et al., Kanyal et al., and Roy et al. the mean years of metformin use were 9.6 ± 6.8 years, 4 years, 2.8 ± 3.3 years, 5±0.8 years, 3.23 ± 1.17 years, and 2.05 ± 0.39 years respectively [24,29-33].

Nearly 45% were taking 1000 mg/day of metformin and just 15% were taking 2 g/day in the present study. In the study by Krishnan et al., 83.9% of patients were taking over 1000 mg of metformin per day [34]. In research conducted by Ko et al., about 62.5% were on a daily metformin dose of 1000 mg, and 12% were on a daily metformin dose of 2000 mg [35]. The study by Chattopadhyay et al. showed that 57% of participants took over 1000 mg to 2 g of metformin daily, and 41% took over 500 mg to 1000 mg daily [27].

In our study, 27% of participants had a deficiency of vitamin B12, and nearly 18% had levels close to the upper limit. In publications by Ahmed et al., Krishnan et al., Baidya et al., and Tal et al., the prevalence was found to be 28.1%, 28.3%, 22%, and 24%, respectively [24,30,34,36]. On the contrary, vitamin B12 deficiency prevalence was lower in studies by Al-Hamdi et al., Romero et al., Nervo et al., Miyan et al., and Ko Set al. in these studies 10.5%, 8.6%, 6.9%, 3.9% and 9.5% respectively was the prevalence recorded [26,31,35,37,38]. The prevalence of vitamin B12 deficiency was higher in studies by Damiao et al., Owhin et al., Raizada et al., and Qureshi et al.; in these studies, the prevalence was 33.3%, 41%, 35.5%, and 33%, respectively [23,39-41].

In our study, vitamin B12 deficiency was significantly associated with age over 45, alcohol intake, diabetes duration of seven years, overweight/obese BMI category, duration of metformin use for seven years, and a daily dose of over 1000 mg. We noticed similar findings in studies by Aroda et al., al-Hamdi et al., Ahmed et al., Krishnan et al., Reinstatler et al., Ko et al., and Raizada et al. in which vitamin B12 deficiency was associated with the duration and daily dose of metformin therapy, respectively, in these studies [12,22,23,30,34,35,37].

Using proton pump inhibitors and H2 blockers, as well as the duration of metformin therapy, was found to be significantly associated with vitamin B12 deficiency in the Damiao et al. study [39]. In the Krishnan et al. study, non-Malay race and diabetes duration were significantly associated with vitamin B12 deficiency [34]. HbA1c, homocysteine levels, metformin duration, and dose were all associated with vitamin B12 deficiency in the Miyan et al. study [26].

In our study, vitamin B12 deficiency was negatively correlated with the duration and daily dose of metformin therapy and positively correlated with hemoglobin values. We observed comparable results in studies by Sato et al., Agarwal et al., Nayyar et al., and Kanyal et al. [25,29,32,42].

Limitations

Our study had a few limitations. First, the study was conducted with a smaller number of samples because of a lack of resources. However, the results might be generalized to a comparable population in a comparable setting. We did not perform nerve conduction investigations and neuropathy screening because of logistical difficulties. A cross-sectional examination of causal relationships and risk estimates is not feasible. A future analytic investigation (case-control or cohort study) in the study population can circumvent this constraint. Patients on combined therapy (metformin plus other medicines) were excluded, which restricts the generalizability of the study. These patients may experience a higher incidence of B12 deficiency and/or neuropathy (or lower).

## Conclusions

According to the findings of the study, a large proportion of the sample group was deficient in vitamin B12. This study also demonstrates that there are gaps in the identification and treatment of vitamin B12 deficiency in the study area, as it is frequently undiagnosed. The longer duration and higher daily dose of metformin were the risk factors for vitamin B12 insufficiency. A vitamin B12 shortage increases the likelihood that diabetic neuropathy may worsen. Therefore, monthly vitamin B12 measurement is required for diabetic patients on long-term, high-dose metformin. Preventative or therapeutic vitamin B12 supplementation can mitigate this issue.
